# Extended-Spectrum-Beta-Lactamase Producing Bacteria Related Urinary Tract Infection in Renal Transplant Recipients and Effect on Allograft Function

**DOI:** 10.1371/journal.pone.0091289

**Published:** 2014-03-17

**Authors:** Poornima Ramadas, Prejith P. Rajendran, Prathik Krishnan, Asha Alex, Eric Siskind, Aditya Kadiyala, Vivek Jayaschandran, Amit Basu, Madhu Bhaskaran, Ernesto P. Molmenti

**Affiliations:** 1 Transplant Program, North Shore University Hospital, Manhasset, New York, United States of America; 2 Department of Surgery, North Shore University Hospital, Manhasset, New York, United States of America; 3 Department of Nephrology, North Shore University Hospital, Manhasset, New York, United States of America; University of Florence, Italy

## Abstract

**Background:**

Urinary tract infection (UTI) is a well-recognized early complication in renal transplant recipients (RTR) and can have significant bearing on their outcome. The recent rise in incidence of extended spectrum beta lactamase (ESBL) producing bacteria causing UTI among RTR poses new and significant challenges in terms of management and outcome. Our aim is to analyze the effect of ESBL producing bacteria causing UTI in these patients and its impact on allograft function.

**Methods:**

We reviewed the medical records of 147 RTR who were followed at a tertiary care hospital affiliated transplant center between January 2007 and May 2013 and noted five RTR who developed episodes of ESBL producing bacteria related UTI during follow up. Multiple patient characteristics including demographics, immunosuppression, recurrences, allograft function and outcome were analyzed.

**Results:**

Five patients (3.4%) out of 147 had ESBL producing bacteria related UTI. We found all patients to be above 60 years of age, with three out of five being females, and all five patients had diabetes mellitus. We identified a total of 37 episodes of UTI among these five patients during this period. Two of these patients had elevated creatinine values during the episodes of UTI and three of them developed bacteremia. Of the five patients, four of them had a favorable outcome except for one patient who developed persistent allograft dysfunction.

**Conclusion:**

RTR are at a higher risk for developing ESBL producing bacteria associated UTI. Early diagnosis along with appropriate and judicious use of antibiotics will ensure long term success in allograft and patient outcome.

## Introduction

Renal transplants have become the standard of care for end stage renal transplant (ESRD), thereby increasing the number of patients on immunosuppressive regimen who are extremely susceptible to a host of infections in the post-transplant phase [1]. Urinary tract infection (UTI) is a common infection in these patients and their effect on the allograft kidney has been well documented in previous studies [2], [3]. Approximately 70% of these UTI's are caused by gram-negative bacteria and can predispose to dehydration, allograft rejection, transplant pyelonephritis and reactivation of Cytomegalovirus (CMV) infection, all subsequently leading to allograft dysfunction and graft failure [4], [5].

We would like to address the issue of extended-spectrum-beta-lactamase (ESBL) producing bacteria causing UTI among renal transplant recipients (RTR) [6]. ESBL is essentially an enzyme located on transferrable bacterial plasmid DNA, capable of hydrolyzing penicillins, extended spectrum cephalosporins as well as monobactams [7]. ESBL is commonly expressed by organisms belonging to Enterobacteriaceae, mainly Escherichia coli and Klebsiella species, both of which are frequently associated with UTI in RTR [6], [7]. RTR have multiple local and systemic risk factors, which predispose them to UTI [8]. Timely intervention and appropriate management of the condition is essential to avoid exacerbation of the clinical course in these patients.

## Subjects and Methods

We reviewed the medical records of 147 renal transplant recipients (RTR) who were followed at a tertiary care hospital affiliated transplant center between January 2007 and May 2013 and noted five RTR who developed episodes of extended-spectrum-beta-lactamase (ESBL) producing bacteria related urinary tract infection (UTI) during follow up. This study was approved by the Institutional review board at North Shore LIJ Hospital, NY. The patient records/information was anonymized and de-identified prior to analysis. The general policy for urinalysis for post-transplant visits at our center is as follows:

First 2 weeks - twice a week

2–4 weeks - once a week

4–12 weeks - once in 2 weeks

3 months-1 year - once a month

1–2 year - once in 2 months

2–5 years - once in 3 months

>5 years - once in 6 months

Clean catch mid-stream urine sample is taken. If there is any evidence of UTI such as leukocytosis or positive nitrites/leukocyte esterase, then a reflex culture is sent. All these patients presented with fever and dysuria and were diagnosed with UTI based on urine microscopy and culture results. Antibiotic sensitivity testing of the cultures was used to confirm the presence of ESBL producing bacteria. All patients were on routine immunosuppression protocol consisting of tacrolimus or cyclosporine, mycophenolate mofetil, and prednisone and prophylactic regimen against bacterial infections consisting of cefazolin 1 gm preoperatively and Trimethoprim-Sulfamethoxazole (80/400) one tab daily life long post-transplant. After the treatment of an episode of UTI, urine cultures are done to confirm resolution of the infection. Subsequent episodes were identified when symptoms of UTI occurred with positive urine cultures for the bacteria. These patients are followed lifelong like other RTR, with concern for the recurrence of UTI. This study was approved by the Institutional review board at this health system.

## Results

Five patients (3.4%) out of 147 had extended-spectrum-beta-lactamase (ESBL) producing bacteria related urinary tract infection (UTI). All patients were above 60 years of age and included 3 female and 2 male patients. Of the two male patients, one was diagnosed with benign prostatic hyperplasia (BPH). Four of them underwent live donor renal transplantation and one received kidney from a deceased donor. All the patients in our study were diabetic. None of them had a ureteral stent inserted in the immediate post-transplant phase. In our study 3 out of 5 patients had episodes of UTI due to non ESBL producing bacteria prior to contracting the ESBL producing one. We identified a total of 37 episodes of UTI among these five patients between January 2007 and May 2013. One of our patients required a ureteral stent later in the post-transplant phase during the course of treatment of urinary stone in the transplanted ureter. None of them were noted to have an episode of rejection. Interestingly two of these patients had elevated creatinine values during the episodes of UTI. One patient was noted to have progressively elevated creatinine during successive episodes of UTI resulting in persistent allograft dysfunction. Of the five patients described in this case study, three developed bacteremia and sepsis. One of them had acute pyelonephritis which progressed into full blown septicemia and shock requiring long term hospitalization and later on developed chronic allograft dysfunction. Carbapenems were the most commonly used treatment in our patients (1 gram ertapenem/meropenem intravenously once daily or 1 gram imipenem intravenously twice daily for 14 days). The potential risk factors we observed in patients other than the genitourinary surgery (kidney transplant) and bladder catheterization were diabetes, prior episodes of non ESBL UTI, ureteral obstruction and ureteral stent, of which all except the prior episodes of non ESBL UTI is applicable to UTI in general.

Refer to Table 1 for details regarding patient characteristics, episodes of UTI, treatment and outcome.

**Table 1:** Patient characteristics, episodes of UTI, treatment and outcome.

	PATIENT 1	PATIENT 2	PATIENT 3	PATIENT 4	PATIENT 5
**AGE (Years)**	62	74	60	73	69
**GENDER**	M	F	F	M	F
**RACE**	Hispanic	Caucasian	Caucasian	South Asian	Persian
**CAUSE OF ESRD**	DM, HTN	DM, HTN	Autoimmune interstitial nephritis	DM, HTN	DM, HTN
**YEAR OF TRANSPLANT**	2011	2011	2010	2006	2005
**TYPE OF DONOR**	Cadaveric	Live	Live	Live	Live
**COMORBIDITIES**	DM, HTN, Hypothyroidism	DM, HTN, Hypothyroidism, Hepatitis C	DM, HTN, Hypothyroidism, CMV viremia	DM, HTN	DM, HTN
**IMMUNOSUPPRESSION PROTOCOL**	Tacrolimus, Mycophenolate mofetil, Prednisone	Tacrolimus, Mycophenolate mofetil, Prednisone	Tacrolimus, Mycophenolate mofetil, Prednisone	Tacrolimus, Mycophenolate mofetil, Prednisone	Cyclosporine, Mycophenolate mofetil, Prednisone
**BASELINE CREATININE POST TRANSPLANT (mg/dl)**	1.62	1.09	0.9	0.8	1.1
**TIME OF FIRST EPISODE OF UTI SINCE TRANSPLANT**	2 weeks	2 weeks	3 months	30 months	36 months
**DETECTION OF ESBL BACTERIA (EPISODE)**	First	Third	Fourth	First	Second
**NO OF UTI RECURRENCES WITH ESBL BACTERIA**	All	4	All	All	5
**OBSTRUCTION**	Stone in the transplanted ureter on CAT scan and ureteral stent inserted. Removed 4 weeks later.	No	No	No	No
**TREATMENT**	Imipenem-cilastatin, Ertapenem (2 weeks)	Pireracillin tazobactam, Meropenem (2 weeks)	Ertapenem (2 weeks)	Imipenem-cilastatin, Ertapenem (2 weeks)	Piperacillin tazobactam, Ertapenem (2 weeks)
**OUTCOME**	Chronic allograft dysfunction	Improved	Improved	Improved	Improved

M-Male, F-Female, ESRD-End stage renal disease, DM-Diabetes mellitus, HTN-Hypertension, CMV-Cytomegalovirus, UTI-Urinary tract infection, ESBL-Extended spectrum beta lactamase.

**Figure 1:**
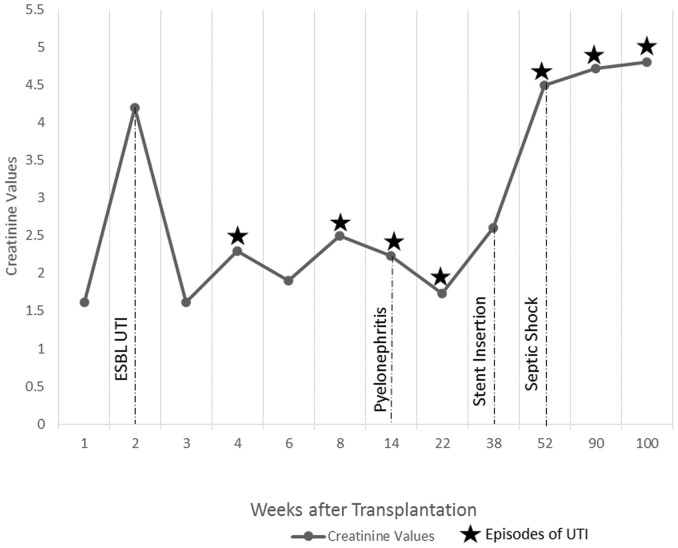
Creatinine Values and Clinical Events of Patient 1.

**Figure 2:**
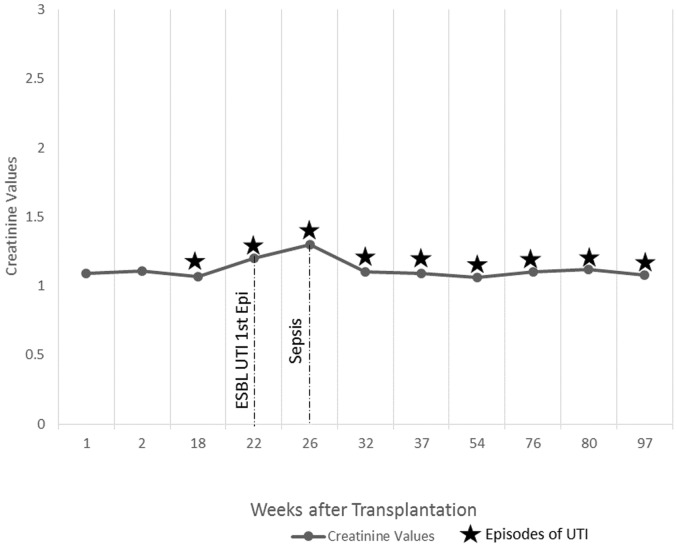
Creatinine Values and Clinical Events of Patient 2.

**Figure 3:**
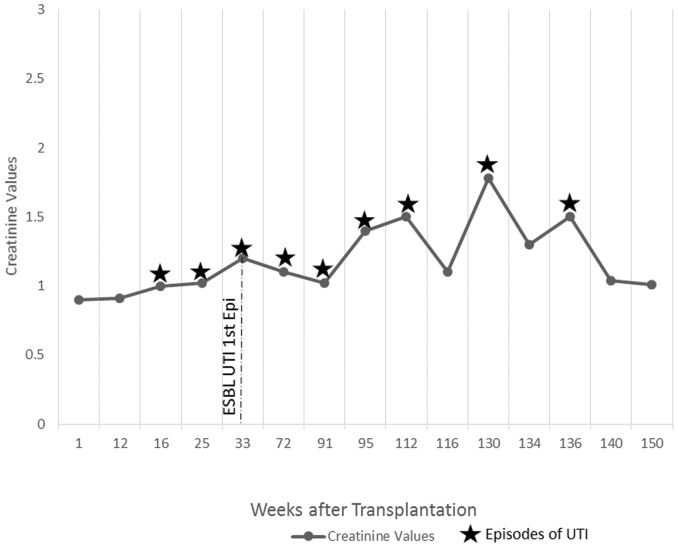
Creatinine Values and Clinical Events of Patient 3.

**Figure 4:**
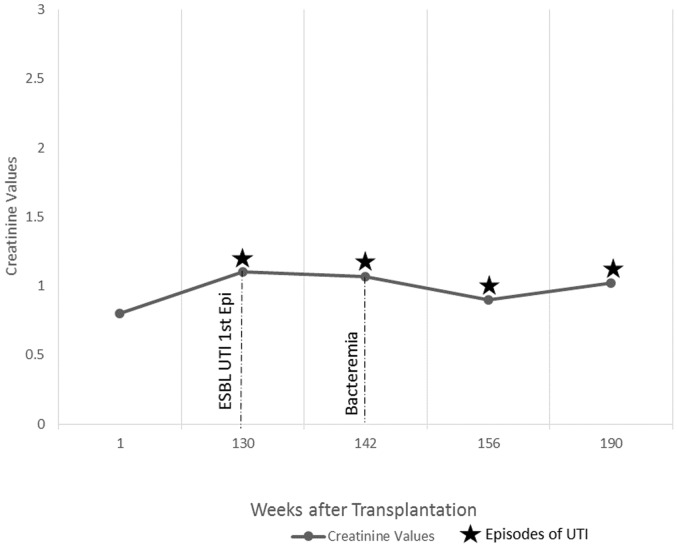
Creatinine Values and Clinical Events of Patient 4.

**Figure 5:**
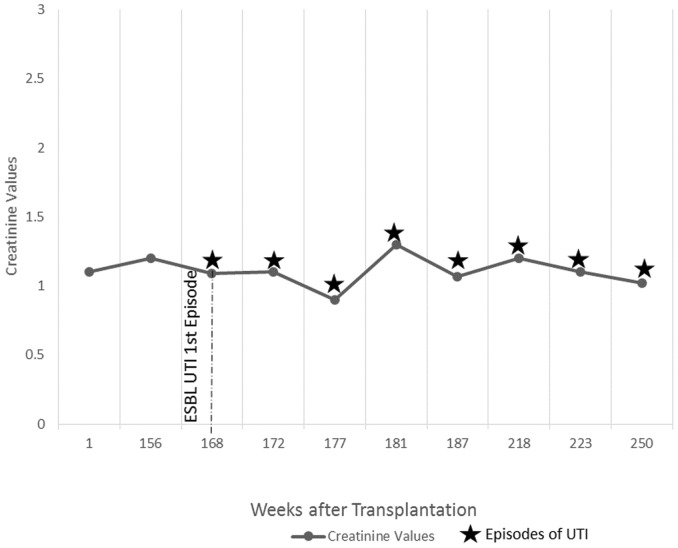
Creatinine Values and Clinical Events of Patient 5.

Refer to Figures 1 to 5 for graphical representation of the creatinine values and the clinical events of the five patients.

## Discussion

Urinary tract infection (UTI) is the most common bacterial infection, representing 40 to 50% of all post-transplant infections [9] and is most commonly seen in the first few weeks following transplantation [4]. Gram-negative bacteria are the culprits in 70 percent of the cases, with E coli being the most common etiology [10]. Antibiotics such as 3rd generation cephalosporins used preoperatively [11] and Trimethoprim-Sulfamethoxazole used in the post-transplant phase have been found to reduce the incidence of UTI [12]. However antibiotic prophylaxis induces bacterial resistance and has led to the emergence of extended-spectrum-beta-lactamase (ESBL) producing bacteria [13]. ESBL resistance has been increasingly reported around the world in recent years. A study in 2010 has shown a 14 percent increase in ESBL bacteria over the time period of 2007–2010 [14]. The choice of antibiotics we have for this condition is limited [15]. In addition ESBL UTI is associated with significant patient mortality and morbidity, which poses a challenge in proper evaluation and management [16].

As in general community acquired UTI, ESBL producing bacterial UTI is associated with numerous risk factors. In our case analysis we focused on age, gender, living or cadaveric donor kidneys, stent insertion during or post transplantation, antibiotic prophylaxis, immunosuppression, diabetes mellitus, rejection, recurrent episodes of UTI and urinary tract obstruction as possible risk factors in this patient population.

There are contradicting data regarding the correlation of age and UTI. A study done in Spain found that age over 60 years is a significant risk factor for ESBL UTI [17]. UTI in elderly patients also have a high risk for septic shock and death [9]. A study at Mayo Clinic in 2009 found that there was no difference in rates of UTI in women over the age of 65 as compared to younger women [18].

It is a well-known fact that female patients have a higher risk of developing UTI, which is mostly due to difference in anatomy [19]. In spite of protective barriers in men, it has been found that ESBL producing bacteria do in fact penetrate this barrier, thus increasing the incidence in males [20].

Studies which correlate the type of donor to the incidence of UTI are rare. A study in 2005 noted an increased incidence in UTI in patients who had cadaveric donors [21].The type of donor is also a major risk factor for chronic allograft dysfunction [22].

Ureteral stents are usually placed intraoperatively to reduce urologic complications post-transplant, the risk of infection being taken into consideration. Studies show that stenting predisposes to UTI and a high recurrence rate of UTI was seen even after stent removal [23]. Early removal of the stent by the end of 2 weeks post-transplant has reduced incidence of UTI compared to late removal [24].

Antibiotic therapy is a significant risk factor for development of ESBL producing bacteria [25]. A study in 2011 suggested an association between UTI and perioperative cephalosporin prophylaxis in renal transplant patients [26].

Immunosuppression is an important risk factor for ESBL UTI. A study done by in 2010 showed a higher incidence of UTI in patients who had cyclosporine in their immunosuppression protocol [27]. Tacrolimus and prednisone have also been associated with an increased risk of infections of which UTI is the most common [28].

Diabetes mellitus predisposes to UTI in general due to decreased local cytokine secretion in the urinary tract and this relationship has been proven by several studies [22]. The reduced local immunity augments the risk of infection by multidrug resistant microorganisms including ESBL producing organisms.

Recurrent UTI can act as predisposing factor for development of ESBL UTI. A study done in 2011 showed over 75% of patients with ESBL had recurrent UTI in the past [29]. Another study done in 2010 also documented increased incidence of ESBL UTI in patients with recurrent UTI [14]. This association could be due to prior use of multiple antibiotics.

Functional or mechanical disorders of urinary tract are other risk factors associated with an increased risk of developing UTI [30]. It has also been found that all patients with vesicoureteral strictures had recurrent UTI [31].

UTI in RTR can have significant negative effects on the allograft kidney and can even lead to allograft failure [32]. UTI can even trigger the onset of acute allograft rejection [33]. UTI is a risk factor for the onset of chronic rejection and requires early and intense treatment [3]. It can also predispose to acute pyelonephritis [34] of the transplant kidney which may progress to systemic bacteremia and septic shock associated with significant morbidity and mortality in these patients [5]. A study using United States Renal Data System Treatment data showed that RTR had a higher incidence of hospitalization due to septicemia, 30.6% of which were secondary to UTI [2].

ESBL producing bacteria related UTI is extremely difficult to treat given the very few drug options available to treat the condition [35], [36]. Two most commonly used antibiotic choices are piperacilin-tazobactam and imipenem [37]. Currently carbapenems are considered as the preferred drug of choice for infections due to ESBL-producing organisms as they are generally resistant to ESBL-mediated hydrolysis [38]. Ertapenem and faropenem are newer carbapenems which are being used more commonly and have excellent activity against ESBL-producing organisms [7]. Fosfomycin, the latest addition has shown to have a high level of broad spectrum antimicrobial activity against ESBL producing enterobacteriacae [39].

## Conclusion

Renal transplant recipients are at a higher risk of developing UTI due to ESBL producing bacteria. As many of the inherent risk factors are non-modifiable, this situation poses a special challenge. Potential adverse consequences of ESBL UTI may include sepsis as well as acute and chronic allograft dysfunction. Efficient management of these patients with a high index of suspicion, prompt diagnosis and use of appropriate antibiotics can result in successful allograft and patient outcome. Continued monitoring on a large scale with close attention to the treatment outcome will help understand and manage this evolving disease.
